# Clinical efficacy and safety of antibiotic combination therapy in the treatment of community-acquired pneumonia in children

**DOI:** 10.12669/pjms.41.4.10236

**Published:** 2025-04

**Authors:** Huan Yang, Zhengke Xiang, Peiwei Chen

**Affiliations:** 1 Huan Yang, Department of Pediatrics, The Central Hospital of Enshi, Tujia and Miao Autonomous Prefecture, Enshi, Hubei, 445000, China; 2 Zhengke Xiang, Department of Pediatrics, The Central Hospital of Enshi, Tujia and Miao Autonomous Prefecture, Enshi, Hubei, 445000, China; 3 Peiwei Chen, Department of Pediatrics, The Central Hospital of Enshi, Tujia and Miao Autonomous Prefecture, Enshi, Hubei, 445000, China

**Keywords:** Azithromycin, Cefotaxime, Children, Community-acquired pneumonia

## Abstract

**Objective:**

To determine the efficacy and safety of cefotaxime combined with azithromycin in the treatment of children with community-acquired pneumonia (CAP) and its effect on inflammatory factors.

**Methods:**

This is an observational study. A total of 118 children with CAP admitted to our hospital from June, 2022 to December, 2023 were randomly selected. According to the random number table method, they were divided into a control group and a combination treatment group, with 59 children in each group. The children in the control group were treated with azithromycin, and those in the combination treatment group were treated with cefotaxime combined with azithromycin. Both groups were treated for a week. The disappearance time of symptoms (fever, pulmonary rales, cough) and the improvement of inflammatory indicators (serum interleukin-6 (IL-6), serum C-reactive protein (CRP), and serum interleukin-8 (IL-8)) after treatment were compared. The clinical efficacy and adverse reactions were compared between the two groups.

**Results:**

The disappearance time of fever, pulmonary rales, and cough in the combination treatment group was significantly shorter than that in the control group (P < 0.05). After treatment, the serum levels of CRP, IL-6, and IL-8 in the two groups were lower than those before treatment, and the reduction in the combination treatment group was larger than that in the control group (P < 0.05). The total efficacy of the combination treatment group was significantly higher than that of the control group (P < 0.05). There was no significant difference in the total incidence of adverse reactions between the two groups (P > 0.05).

**Conclusion:**

Cefotaxime combined with azithromycin in the treatment of CAP in children can significantly improve the efficacy, quickly relieve clinical symptoms, improve the body’s defense mechanism, inhibit inflammatory response, and has high safety, which is worthy of clinical promotion.

## INTRODUCTION

Community-acquired pneumonia (CAP) in children is a common pediatric respiratory disease, mainly manifested as fever, cough, sputum, and chest pain in clinics, and the common early symptoms include nasal congestion, runny nose, sneezing, sore throat, and headache.^1^,^2^ If the treatment for childhood community-acquired pneumonia (CAP) is not timely or improper, it can cause inflammation in the lungs, leading to impaired gas exchange and potential complications such as shock, pulmonary heart disease, respiratory failure, and a higher risk of death.^3^,^4^

This disease is one of the leading causes of death in children younger than five years of age. According to the World Health Organization (WHO), about 922,000 children worldwide died from CAPin 2015.^5^ CAP in children is mainly caused by bacterial or viral infections such as *Streptococcus pneumoniae, Staphylococcus, and Haemophilus influenzae*, and its treatment is generally based on antibiotics, supplemented by cough, phlegm, and other symptom relieving drugs.^6^,^7^ Azithromycin is a broad-spectrum antibiotic, which has good pharmacokinetic and pharmacodynamic properties in clinical application and has high antibacterial activity against a variety of bacteria such as *Streptococcus pneumoniae, Haemophilus influenzae, and Mycoplasma pneumoniae*, but its widespread use will lead to increased bacterial resistance.^8^

In order to deal with the problem of bacterial resistance to azithromycin, some authors suggest that the combination of azithromycin and cephalosporin antibiotics should be used to treat CAP in children.^9^ Cefotaxime is a kind of cephalosporin antibiotic, which has high antibacterial activity against a variety of bacteria and high sensitivity to some drug-resistant strains.^10^ A Chinese study has shown that the use of azithromycin combined with cefotaxime in the treatment of children with CAP can achieve good therapeutic effect.^11^ The purpose of this study was to explore the clinical efficacy of azithromycin combined with cefotaxime in the treatment of CAP in children to provide scientific theoretical basis and support for the clinical medication of CAP in children. The report is as follows.

## METHODS

A total of 118 children with CAP admitted to the Department of Pediatrics in the Central Hospital of Enshi, Tujia and Miao Autonomous Prefecture from June 2022 to December 2023 were randomly selected and divided into control group and combination treatment groups according to random number table method, with 59 in each group. All the children were admitted to the general pediatric ward.

In this observational study the calculation formula of the sample size is:


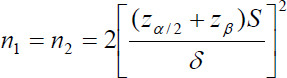


where n_1_ and n_2_ are the content that are needed by two samples, S is the estimated value of the two overall standard deviations, and δ = μ_1_-μ_2_, which is the difference of two mean values.

### Inclusion criteria:

- Children diagnosed with CAP according to the Guidelines for Management of Childhood Community Acquired Pneumonia (revised in 2013) (Part 1)^12^ (diagnostic criteria: cough occurs or the symptoms of the original respiratory diseases worsen; fever occurs with a body temperature higher than 38°C in the early stage of the disease, and the duration is uncertain; symptoms such as polypnea and dyspnea occur; chest X-ray examination shows patchy or sheet-like pulmonary shadows; the white blood cell count is elevated).
- Children aged six months to 13 years (include 13 years).
- Family members of the children signed informed consent (No.EYLL-2022-0623 dated on May 25^th^ 2022).

### Exclusion criteria:

- Severe CAP.
- Patients with congenital underlying diseases such as heart disease, airway malformation, and immune deficiency.
- Accompanied by other serious complications such as severe extrapulmonary complications and respiratory failure.
- Transfer to another hospital or discharge automatically.
- A history of allergy to therapeutic drugs.

### Treatment methods:

Children in control group were given azithromycin injection (Yabao Pharmaceutical Group Co., LTD., China) at a dosage of 10 mg/kg intravenously, once a day. The children in the combination treatment group were given cefotaxime sodium for injection (Shandong Lukang Pharmaceutical Co., LTD., China) at a dosage of 50 mg·kg^-1^·d^-1^ through intravenous infusion, twice a day, in addition to azithromycin injection (10 mg/kg intravenously, once a day). The treatment lasted for one week in both groups.

### Ethical Approval:

This study was reviewed and approved by the Ethics Committee of the hospital (Ref. No.: EYLL-2022-0623, dated May 25, 2022).

### Observational indicators and efficacy criteria:

- The disappearance time of symptoms and signs such as fever, cough, and moist rales (symptoms or signs disappear for at least 48 hours);
- Inflammatory indicators: 3 ml of fasting venous blood was collected from the children in the morning before treatment and one week after treatment, and serum was obtained. Serum interleukin-6 (IL-6), serum interleukin-8 (IL-8), and C-reactive protein (CRP) were determined by immunoturbidimetry. The detection kit was purchased from Weike Inspection Group (Suzhou) Co., LTD.
- Clinical efficacy: The criteria of efficacy was evaluated according to Guidelines for the Diagnosis and Treatment of Mycoplasma pneumoniae pneumonia in Children (2023).^13^

The efficacy can be divided as: significantly effective - symptoms and signs of children significantly improve, and X-ray results show completely absorbed lesion after treatment; effective - the symptoms and signs of the children are improved to a certain extent, and the lesion is basically absorbed under X-ray examination after treatment; invalid - no improvement or even aggravation of symptoms and signs after treatment, and no significant change or even aggravation of lesions under X-ray examination. The total efficacy after treatment was calculated: the total efficacy = (number of significantly effective cases + number of effective cases)/total number of cases ×100%. (4) Adverse reactions included rash, nausea, vomiting, and diarrhea.

### Statistical analysis:

SPSS22.0 statistical software package was used for processing. Quantitative data were represented by (Mean±SD), all of which were in line with normal distribution, and comparison was performed by t test. Qualitative data was represented by n (%), and comparison was performed by chi square test. P < 0.05 indicated a statistically significant difference.

## RESULTS

There was no significant difference in general data between the two groups (P > 0.05), as shown in Table-I. The disappearance time of fever, rales, and cough in the combination treatment group was earlier than that in the control group, showing a statistically significant difference (P < 0.05) (Table-II). Before treatment, there was no significant difference in serum IL-6, IL-8, and CRP levels between the two groups (P > 0.05). After one week of treatment, the levels of these indicators in both groups were lower than before treatment, and the combination treatment group was lower than the control group (P < 0.05) (Table-III).

**Table-I:** Comparison of general data between the two groups.

Group		Combination treatment group	Control group	t/X^2^	P
Age (Mean±SD, years)		7.26±1.02	7.24±0.88	0.973	> 0.05
Gender [n (%)]	Male	33	35	0.157	> 0.05
	Female	26	24	
Duration of disease (Mean±SD, d)		7.37±1.30	7.38±1.55	0.118	> 0.05

**Table-II:** Comparison of symptom disappearance time between the two groups (Mean±SD, d).

Group	Combination treatment group	Control group	t	P
Fever	1.87±1.79	2.95±2.46	1.850	P<0.05
Moist crackles	4.53±2.63	6.81±3.37	2.816	P<0.05
Cough	5.64±1.17	7.84±1.93	5.034	P<0.05

**Table-III:** Comparison of serum inflammatory factor levels between the two groups (Mean±SD, pg/mL).

Group		Combination treatment group	Control group
IL-6	Pre-treatment	368.26±16.45	372.83±26.93
	After treatment	94.27±16.11*^#^	103.89±19.03
IL-8	Pre-treatment	18.66±2.65	18.04±2.96
	After treatment	10.36±4.55*^#^	12.65±5.02
CRP	Pre-treatment	246.03±30.81	249.21±24.86
	After treatment	56.33±10.96*^#^	60.24±23.32

Note:

*: compared with before treatment, P < 0.05;

#: compared with control group, P < 0.05.

The total efficacy of the combination treatment group was 96.61%, which was significantly higher than that of the control group (77.97%, P < 0.05) (Table-IV). There was no significant difference in the total incidence of adverse reactions between the two group (P > 0.05), as shown in Table-V.

**Table-IV:** Comparison of clinical efficacy between the two groups [n (%)].

Group	Combination treatment group	Control group	X^2^	P
Significantly effective	39 (66.10)	22 (37.29)	/	/
Effective	18 (30.51)	24 (40.68)	/	/
Ineffective	2 (3.39)	13 (22.03)	/	/
Total efficacy	57 (96.61)	46 (77.97)	4.102	P < 0.05

**Table-V:** Comparison of adverse reactions between the two groups [n (%)].

Groups	Combination treatment group	Control group	X^2^	P
Rash	1 (1.70)	2 (3.40)	/	/
Nausea and vomiting	0 (0.00)	1 (1.70)	/	/
Diarrhea	1 (1.70)	2 (3.40)	/	/
Total incidence	2 (3.40)	5 (8.50)	2.216	P > 0.05

## DISCUSSION

The results of this study showed that compared with the control group, the combination treatment group had higher total efficacy and earlier disappearance of symptoms, which was similar to the results of previous domestic studies.^14^,^15^ In the study of Zhong et al.^16^, it was also found that the hospitalization time and the duration of symptoms such as cough, fever, and pulmonary rales of the children treated with azithromycin combined with cefotaxime sodium were shorter than those of the reference group. The research results of Li et al. also clearly confirmed that the clinical efficacy of children treated with azithromycin combined with cefotaxime sodium was better than that of the control group treated with conventional drugs.^17^ However, the total efficacy of treatment in the observation group in this study was slightly lower than that in this study. It is considered that it may be related to individual differences of patients. The above research results all suggest that cefotaxime combined with azithromycin can expand the antibacterial spectrum and improve the antibacterial effect, and the combined application has a broader coverage of pathogens and can exert a good antibacterial effect.^18^,^19^

As pro-inflammatory factors, IL-6 and IL-8 can regulate the immune system and aggravate the inflammatory response.^20^ CRP will activate leukocyte phagocytosis when the body has inflammation. The results of this study showed that serum inflammatory factors were significantly reduced in the two groups after treatment, and the reduction range in the combination treatment group was greater than that in the control group. In the study by Huang et al.^21^, inflammatory indicators were also included for observation. Although the inflammatory indicators they studied were somewhat different from those in this study, the results also found that the levels of serum inflammatory factors in the observation group after treatment were lower than those in the control group, suggesting that cefotaxime combined with azithromycin can reduce the pulmonary inflammatory response in children with CAP, can reduce the degree of bacterial infection, effectively inhibit the inflammatory response, and prevent the continuous deterioration of the disease.

In terms of safety, the results of this study showed that the incidence of adverse reactions in the combination treatment group was 3.4%, which was not significantly different from 8.5% in the control group. It indicated that the interaction of azithromycin and cefotaxime was relatively stable and did not increase adverse reactions. In the study by Deng et al.^22^, the control group was treated with azithromycin, and the observation group was treated with the combined treatment of azithromycin and cefotaxime sodium. The results also showed that there was no significant difference in the incidence of adverse reactions between the two groups. However, none of the 70 patients in the above study had a rash. Therefore, regarding the types of symptoms of adverse reactions, it is necessary to expand the sample group for further research.

Although there have been studies on the treatment of CAP with cefotaxime combined with azithromycin, the conclusions drawn from the previous related studies are inconsistent, and there is a lack of observation subjects such as infants. In most studies, the included subjects are children over nine months old. In this study, children with CAP aged six months to 13 years were taken as the case group, and a control group was also established, which just supplemented the analysis of the therapeutic effect on younger infants. This has important guiding significance for clinical practice.

### Limitations:

This study did not analyze the cost-effectiveness of the two medication regiments for the children, nor did it follow up the recurrence situation. Moreover, the sample size was small. In the future, the number of observation subjects can be increased according to the actual situation, other laboratory tests that are instructive for diagnosis and treatment can be improved, multi-center studies can be carried out, and relevant experimental studies can be actively conducted to serve and guide clinical practice better.

## CONCLUSION

Azithromycin combined with cefotaxime sodium can effectively improve the therapeutic effect for CAP in children. It can improve the body’s defense mechanism and inhibit inflammatory response, while maintaining a high level of safety.

### Authors’ Contribution:

**HY:** Study design, Literature search, data collection and analysis.

**ZX** & **PC:** Manuscript preparation, drafting and revising.

**HY** & **PC:** Critical Review and final approval of manuscript.

All authors have approved the final version and are accountable for the integrity of the study.
